# Atypical Presentation of Mixed Connective Tissue Disorder Involving Bilateral Diaphragm

**DOI:** 10.7759/cureus.22154

**Published:** 2022-02-12

**Authors:** Sailaja Devi Saragadam, Srikanth Mukkera

**Affiliations:** 1 Department of Internal Medicine, Texas Tech University Health Sciences Center at Permian Basin, Odessa, USA; 2 Department of Internal Medicine, Texas Tech University Health Science Center at Permian Basin, Odessa, USA

**Keywords:** diaphragm muscle, congestive heart faiulre, severe respiratory failure, diaphragm dysfunction, mixed connective tissue disease

## Abstract

Pulmonary manifestations can be present in 20-80% of patients having mixed connective tissue disorder (MCTD) and are usually subacute. MCTD when associated with polymyositis can rarely involve the diaphragm, causing respiratory failure.

We present herein the case of a 49-year-old female having MCTD with a component of polymyositis who presented with bilateral diaphragmatic paralysis followed by heart failure requiring respiratory support with non-invasive mechanical ventilation. We are aware of only one prior instance of MCTD associated with unilateral diaphragmatic weakness causing mild respiratory dysfunction. To the best of our knowledge, this is the second reported case of diaphragmatic involvement in the MCTD population, with bilateral diaphragmatic paralysis causing severe respiratory failure. This is also the first reported case of such an unusual initial presentation in this patient group. Pulmonary involvement has a poor prognosis. Early diagnosis with the initiation of therapy can improve mortality outcomes in this patient population.

## Introduction

The definition of MCTD is constantly evolving. Currently, the incidence of MCTD is about two persons per 100,000 person-years [1]. However, due to the ambiguity in the available clinical guidelines and criteria, the median time taken to diagnose MCTD after the presentation of the first symptom is almost three and a half years [1]. Pulmonary complications can further delay the diagnosis if they are the initial presenting symptom as they are usually subclinical. Early recognition of these complications by screening for them in our daily clinical practice is important for timely management and in preventing mortality associated with them. Currently, symptomatic management remains the mainstay of treatment in these patients. Though steroids and immunosuppressive therapy are commonly prescribed, their benefit is not clearly established. Further studies need to be done to understand the pathological process of these pulmonary phenomena to develop targeted drug therapy. In our case report, we discuss the initial presentation, diagnostic workup done, and the current data available in the management of such patient populations.

## Case presentation

**Figure 1:**
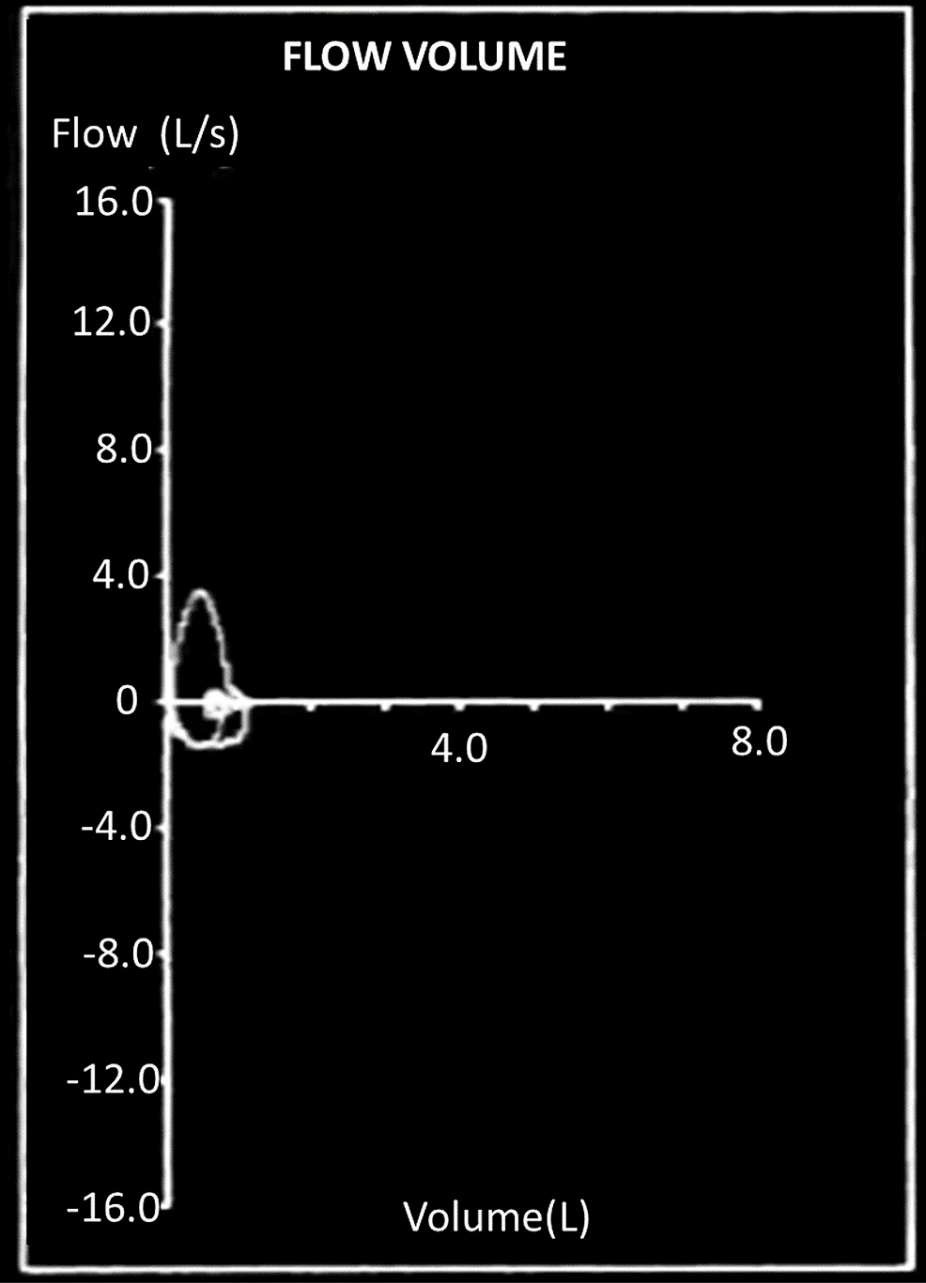
Flow volume loop Flow volume loop showing restrictive disease pattern

A 49-year-old lady with a history of multiple hospital admissions for chronic obstructive pulmonary disease exacerbation (COPD) and hypercarbic respiratory failure requiring to be treated with noninvasive mechanical ventilation (NIV), presents to the clinic for evaluation of an abnormal autoimmune panel. Pulmonary function tests (Figure 1, Table 1) showed a restrictive pattern in contrast to her COPD history. Computed tomography (CT) of the thorax with contrast was negative for the presence of pulmonary embolus, mediastinal, hilar lymphadenopathy, and interstitial lung disease.

**Table 1:** Pulmonary function test done in both supine and sitting position Note though the FEV1/FVC ratio represents restrictive disease, the mild reduction in DLCO can be explained by underlying COPD. There is also a reduction in the FVC predicted in supine when compared to sitting position, demonstrating signs of developing neuromuscular dysfunction. FVC: forced vital capacity, FEV: forced expiratory volume, FEF: forced expiratory flow, RVC: relative volume change, DLCO: Diffusing capacity of the lungs for carbon monoxide, MIP: maximal inspiratory pressure, MEP: maximum expiratory pressure, MVV: maximum voluntary ventilation

	UNITS	Predicted (Sitting)	Pre drug reported (Sitting)	Pre drug % predicted (Sitting)	Predicted (Supine)	Pre drug reported (Supine)	Pre dug % predicted (Supine)
FVC	L,btps	3.23	1.09<	34<	3.23	0.87<	27<
FEV1	L,btps	2.67	0.91<	34<	2.67	0.77<	29<
FEV1/FVC	%	82	83	101	82	88	108
FEV3	L,btps	3.09	0.99	32	3.09	0.83<	27<
FEV3/FVC	%	95	91	95	95	96	100
FEF25-75%	L/s	2.94	1.42<	48<	2.94	2.09	71<
FEF25%	L/s		2.25			3.01	
FEF50%	L/s		1.98			2.65	
FEF75%	L/s		0.52			0.85	
FEFmax	L/s	6.71	2.28	52<		3.13	
RVC	L,btps		1.13			0.82	
RF50%	L/s		1.55			1.24	
DLCO	ml/mmhg/min	28.12	16.0	57			
Plmax/MIP	cm H2O		50			40	
Pemax/MEP	cm H2O		52			32	
MVV	L/min,btps	96.29			96.29	

**Figure 2:**
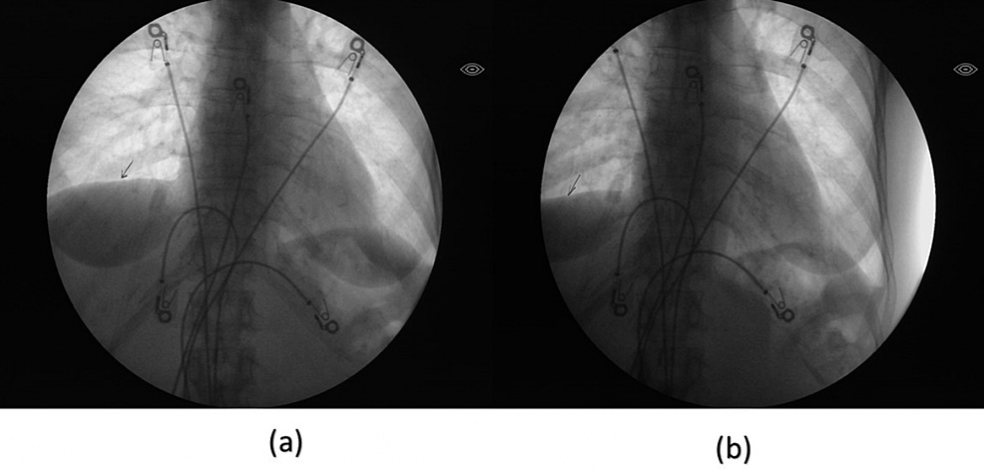
Diaphragmatic fluoroscopy Image (a) and (b) are taken before and after deep inspiration respectively. Note very minimal change in the position of the diaphragm with respect to ribs and heart, more pronounced on the right when compared to the left representing partial bilateral diaphragmatic paralysis.

Diaphragmatic fluoroscopy (Figure 2) confirmed bilateral diaphragmatic paralysis making her dependent on continuous NIV for adequate respiratory support. In the later hospitalizations, as her B-type natriuretic peptide (BNP) was elevated and chest x-ray showed signs of pulmonary edema, an echo was done which showed left ventricular ejection fraction (LVEF) 30-40% with normal RVSP (right ventricular systolic pressure) and moderate to severe global hypokinesis. Left heart catheterization was negative for coronary artery disease suggesting nonischemic pathology for the heart failure. 

On review of symptoms at our clinic, the patient complained of myalgia, polyarthralgia involving both small and large joints which started shortly after the hospital admissions for respiratory and heart failure. The patient had duodenojejunostomy for superior mesenteric artery stenosis a year prior to the initial hospitalization. She denied any family history of autoimmune and neuromuscular disease. The patient was a former smoker with thirty pack-years smoking history and quit in 2019 and drinks alcohol socially. On examination, no dermatological manifestations were noted and the muscle strength of proximal muscle groups in the bilateral extremities was mildly reduced along with reduced bilateral breath sounds on lung auscultation. No other neurological deficit was noted.

The autoimmune panel was positive for ANA (antinuclear antibody) ab test (1:2560 titers - homogenous pattern) with elevated ribonucleoprotein (RNP) Ab >8.0 (0.0-0.9), anti-U1 ribonucleoprotein (U1RNP) 58 (<20), and anti-Sjögren's-syndrome-related antigen A autoantibodies (SS-A 52kD Ab IgG) - 39 (<20). Erythrocyte sedimentation rate (ESR), creatine kinase (CK), and aldolase levels were also elevated. The rest of the autoimmune panel was negative. Nerve conduction studies (NCS) were normal. Electromyography (EMG) (Table 2) revealed widespread myotonic discharges in selected proximal muscles combined with a positive sharp wave and fibrillation potentials indicative of muscular dysfunction. Muscle biopsy showed necrotizing myopathy with inflammation. 

**Table 2:** Electromyography studies showing the presence of + ve positive sharp waves, fibrillations, and complex repetitive discharges PSW: positive sharp waves, Fibs: fibrillation, Fasc: fasciculation, CRD: complex repetitive discharges, Amp: amplitude, Dur: duration, N: normal

Electromyography			Spontaneous				Exertional (MUAP)			
Side	Muscle	Insert Activity	PSW	Fibs	Fasc	CRD	Amp	Dur	Phases	Recruit
R	Triceps	N	Sust	2+	0	2+	-	1+	2+	N
R	Deltoid	N	Sust	2+	0	2+	-	2+	2+	N
R	Tibialis Anterior	N	Unsust	1+	0	3+	-	3+	3+	N
R	Vastus Medialis	N	0	0	0	1+	N	1+	1+	N
R	Gluteus Medius	N	Unsust	0	0	2+	-	2+	2+	N
L	Tibialis anterior	N	Sust	1+	0	3+	-	3+	3+	N

She was diagnosed with MCTD with components of systemic lupus erythematosus (SLE) and polymyositis based on the combination of her clinical history with her autoimmune panel and biopsy results. She was started on intravenous immune globulin (IVIg) therapy for four days along with a steroid tapered dose. She was placed on maintenance therapy with hydroxychloroquine 200mg per os (PO) daily, methotrexate 12.5 mg PO weekly along with goal-directed therapy for heart failure, and continuous positive airway pressure (CPAP) for respiratory support.

Over the next six months, the patient did not have any further hospital admissions and the inflammatory markers started trending down however, her oxygen requirements continued to remain the same suggesting no improvement in the diaphragmatic paralysis. The patient is scheduled to receive maintenance IVIg treatment every six months.

## Discussion

Mixed connective tissue disorder (MCTD) is an immune-mediated disorder presenting with high titers of speckled antinuclear antibodies (>1:1000), serum anti-ribonucleoprotein (RNP) antibodies, RNase- sensitive extractable nuclear antigen antibodies (ENA), and clinical features overlapping with two or more autoimmune rheumatologic diseases like systemic lupus erythematosus, polymyositis/dermatomyositis, systemic sclerosis, rheumatoid arthritis, etc. The most common initial presentation is Raynaud's phenomenon (about 50%) followed by polyarthralgia involving both larger and smaller joints (30%) and swollen hands (16%) [1]. The occurrence of pleuropulmonary manifestations as the initial presenting symptom is rare and when they do present, the most common presentations include interstitial lung disease (ILD), pulmonary hypertension, pleural effusion, pulmonary thromboembolism, and vasculitis [2]. 

However, when MCTD is associated with polymyositis, the respiratory muscles such as the diaphragm can be involved leading to respiratory distress as in our patient. Obtaining detailed clinical history with complete physical examination is essential to identify the underlying autoimmune pathology and order the necessary diagnostic tests. Due to severe respiratory muscle weakness, these patients show restrictive lung disease patterns on pulmonary function tests with reduced lung volumes, maximal inspiratory pressures and expiratory pressures, and increased residual volume. The ratio of forced expiratory volume in one second and vital capacity (FEV1:VC) is usually normal. These patients also demonstrate reduced lung volumes on chest imaging with diaphragmatic elevation. They are at high risk of pneumonia due to weak cough reflex and difficulty in clearing mucous secretions like in our patient leading to recurrent hospitalizations [3].

Diaphragmatic paralysis secondary to recent surgical complications cannot be completely ruled out in our patient however, the presentation timeline with an asymptomatic period in between along with diagnostic findings make this an unlikely cause. To date, four diagnostic criteria have been developed for MCTD. Studies have demonstrated both Alarcon-Segovia and Kasukawa criteria to be equally sensitive (72%), Kahn and Sharp criteria to be less sensitive, around 54% and 28% respectively [1]. Our patient has met Kasukawa et al criteria for MCTD with components of SLE and polymyositis. This diagnosis was further supported by the patient’s positive autoimmune evaluation, EMG studies, clinical history, negative NCS studies combined with histopathological findings of peripheral muscle biopsy. 

Although diaphragmatic biopsy can help in confirming the diagnosis of MCTD when involved, it is seldomly done due to the technical difficulty in obtaining one. Currently, pulmonary function tests, diaphragmatic fluoroscopy along with muscle biopsy, and neuromuscular studies (NMS) studies are used to support the clinical diagnosis [4]. In the case, reported by J. Martens and M. Demedts, the diaphragmatic dysfunction was benign and was diagnosed on routine evaluation. After treatment with steroids, the respiratory symptoms of the patient remained stable suggesting benign diaphragmatic myopathy, unlike our patient [5].

Currently, management of symptoms with respiratory support remains the standard of care in these patients. Although pulmonary complications like pleurisy and acute inflammatory lung processes have shown a positive response to a combination of steroid and immunosuppressive therapy, its benefit in the treatment of respiratory failure from diaphragmatic muscle dysfunction is not well established [6]. This emphasizes the need for large-scale studies to evaluate the effectiveness of these drugs.

## Conclusions

MCTD can present with respiratory failure as the initial presenting symptom. If the diaphragm is involved, diaphragmatic biopsy confirms the diagnosis however diaphragmatic fluoroscopy with neuromuscular studies and proximal muscle biopsy can help in supporting the diagnosis. Though steroids and immunosuppressive therapy have been clinically tried in our current case and past reports, the results are conflicting. Large-scale clinical studies are needed to establish their benefit in this patient population
